# Sexual and reproductive health in young women with ADHD from the view of health care professionals

**DOI:** 10.1186/s12905-024-03230-9

**Published:** 2024-07-05

**Authors:** Karin Wallin, Siw Alehagen, Lena Hanberger, Inger Wallin Lundell, Sally Hultsjö

**Affiliations:** 1 Department of Obstetrics and Gynecology in Linköping, Department of Health, Medicine and Caring Sciences, Linköping University, Linköping, Sweden, 581 83 Linköping, SE Sweden; 2 Department of Health, Medicine and Caring Sciences, Linköping University, Linköping, Sweden, 581 83 Linköping, SE Sweden; 3 Department of Health Sciences, Swedish Red Cross University, Huddinge, Sweden; 4 Department of Psychiatry, Ryhov County Hospital, Jönköping, Sweden

**Keywords:** ADHD, Health care professionals, Sexual and reproductive health, Qualitative method, Young women

## Abstract

**Background:**

Sexual risk-taking and struggles in managing romantic relationships may put young women with Attention Deficit Hyperactivity Disorder (ADHD) at risk of sexually transmitted diseases, unplanned pregnancies, and low relational satisfaction. To gain understanding of sexual behaviors and intimate relationships, this study aimed to identify and describe health care professionals’ (HCPs) perceptions and experiences of sexual and reproductive health (SRH) in young women with ADHD.

**Methods:**

Qualitative interviews were performed with 16 HCPs. Data was analyzed using reflexive thematic analysis.

**Results:**

Analysis resulted in the themes *Struggling to meet expectations, Sexual risk-taking*, and *Complex romantic relationships.* HCPs’ perceptions and experiences indicated that some women were afraid to be judged in clinical meetings when not living up to perceived expectations of sexual behaviors. Lack of impulse control was interpreted by HCPs to result in risk-taking behaviors leading to both negative and positive sexual experiences. Difficulties in assessing intentions of sexual partners were further perceived by HCPs to sometimes lead to sexual regrets or sexual victimization. The HCPs had experience of women wishing for romantic relationships but described these as being complicated by previous experiences, low self-esteem and conflict. ADHD medication and self-knowledge were perceived by HCPs to facilitate the women’s relationship quality.

**Conclusions:**

This study highlights that, from the perspective of HCPs, self-stigmatization and hesitation to raise issues concerning sexuality with HCPs may pose risks for young women with ADHD. It provides insight into sexual risk-taking behaviors, showing the link to regretted sex and sexual victimization. The study concludes that there is a need for HCPs to understand the influence of stigma concerning ADHD and female sexuality as well as how symptoms and outcomes of living with ADHD may impact SRH in order to promote healthy behaviors and relationships in young women.

## Background

Young women with Attention Deficit Hyperactivity Disorder (ADHD), may be at risk of compromised sexual and reproductive health (SRH), thus not receiving fulfillment of the human right to have safe pleasurable sexual experiences free of stigma and coercion, relationships built on trust and communication, and the possibility to make decisions governing one’s own body [1].

ADHD is a neuropsychiatric disorder affecting approximately 3% of adults [2]. The diagnosis is characterized by cognitive deficits in self-regulation of attention, level of activity and impulse control causing impairment of daily function across the lifespan [3]. Comorbid conditions like Autism Spectrum Disorder (ASD), personality disorders, substance use disorders and anxiety and depressive disorders are common [4]. Gender differences in the presentation of ADHD symptoms and symptoms of comorbid diagnoses make women less likely to be referred for assessment, delaying diagnosis and treatment [5].

Young adulthood is characterized by an interest in exploring sexuality and it is common to engage in both causal sexual relationships and romantic relationships [6]. Exploration is a natural part of human development, influenced by biological and emotional aspects such as sexual desire and the wish to form bonds of deeper feelings with others. Sexual development is also dependent on societal attitudes and cultural norms concerning sexuality [7]. In women with ADHD, low self-esteem and difficulties with impulse control and emotion regulation, have been seen to challenge sexual exploration and engagement in intimate relationships [5].

Compared with young adults without a diagnosis, research has shown that young women with ADHD more often engage in sexual risk-taking behaviors such as not using a condom, early sexual initiation and having several sexual partners [8, 9]. It is also more common for them to experience unplanned pregnancy, sexually transmitted diseases (STDs), and sexual victimization [10–12]. Even though temporary sexual relationships being common, young women with ADHD have reported fewer romantic relationships and lower satisfaction with relationships and sexual life compared to men with a diagnosis [13, 14]. According to Marsh et al. [15], higher levels of ADHD symptoms were related to greater fear of intimacy and lower levels of relational self-competence in young adults with ADHD. Low self-esteem was further described by Wallin et al. [16] to make women with ADHD less secure about communicating wishes and desires to a romantic partner, influencing their ability to experience sexual pleasure on equal terms.

This study was conducted in Sweden, where young adults can visit specialized HCPs in youth clinics that are free of charge, or gynecological clinics for counseling and treatment concerning SRH [17]. Assessment of ADHD, follow-up medical treatment, and individualized counseling to manage everyday life, on the other hand, are carried out in primary health care clinics or outpatient psychiatric clinics [18]. Considering that sexuality is an integral part of life, it is possible that the women with ADHD also seek counseling regarding sexual behaviors and relationships within psychiatric care. To gain a broader understanding of SRH in young women with ADHD, we found it important in this study to capture perceptions and experiences from HCPs in various professions specializing in both SRH and ADHD.

HCPs working in youth clinics, gynecological clinics and psychiatric clinics regularly encounter young women with ADHD, accumulating knowledge about SRH in this group. However, the study by Klink Carlander et al. [19] is one of few studies that have looked at the HCPs’ perspective of SRH in young women. HCPs in the study described that it was challenging to find suitable contraceptives and highlighted how communication difficulties and missed appointments could lead to insufficient counseling. Learning from HCPs’ perceptions in our study could supplement the relatively limited research concerning SRH in young women with ADHD and provide a better understanding of the women’s motivations for sexual behaviors, and underlying reasons for challenges in romantic relationships. Becoming aware of HCPs’ views may also help with understanding how to improve health encounters and individualize counseling for the women, promoting healthy sexual behaviors and relationships.

Thus, the aim of this study was to identify and describe health care professionals’ perceptions and experiences of sexual and reproductive health in young women with ADHD.

## Methods

The study had a qualitative inductive design with a latent approach. Data was analyzed using reflexive thematic analysis [20, 21]. The design was found appropriate for exploring perceptions and experiences in a relatively unknown research area, as it allows new insights to appear [22].

### Participants

HCPs with experience of professional encounters with young women with ADHD relating to SRH were included in the study. A purposive sampling of HCPs was used, selected from three psychiatric clinics, two gynecological clinics and three youth clinics specializing in SRH. The clinics belonged to a university hospital, three central hospitals and public health care (youth clinics), situated in the south and south-east regions of Sweden. HCPs received written information about the study by e-mail, and informed consent to participate was given by 18 health professionals. Two HCPs withdrew their wish to participate due to a high workload. All participants were female. None of them had a previous or ongoing professional relationship with any of the researchers. Their profession, age and time of work experience varied, see Table 1.

**Table 1:** Characteristics of HCPs (*n* = 16)

**Median age in years (min-max)**		42 (24–64)
**Median years in profession (min-max)**		12.5 (2–40)
**Profession**		
Midwife		7
Occupational therapist		1
Psychiatric nurse		4
Psychologist		2
Social worker		2

### Data collection

Data collection started with individual interviews. Due to the COVID-19 pandemic, participant recruitment was paused several times. To manage the data collection within a reasonable time frame, focus groups were also offered. In total, eight individual interviews and two focus groups with four participants in each group were conducted. The first focus group included HCPs from a psychiatric clinic (three psychiatric nurses and one occupational therapist), while the second group consisted of HCPs from a youth clinic (three midwives and one social worker). All 16 participants were informed of the voluntary nature of the study and the possibility to withdraw at any time without explanation. To create a safe environment where they could feel free to express themselves, the participants chose time and place for the interview [23]. KW conducted all interviews face to face or using a video link. During focus groups, KW facilitated discussions, assisted by either SH or IWL, who observed non-verbal interaction and group dynamic and documented the general content of the interviews. Supplemented observations were used in data analysis. Due to the COVID-19 restrictions, the first focus group was held via video link with the participants gathered in a conference room and researchers visible on a big screen. In the second group researchers and participants met in person. HCPs were asked to only describe perceptions and experiences of SRH based on encounters with women with a recorded diagnosis or where women talked about their diagnosis during their visit. The same semi-structured questions were used in all interviews. Questions were not covered in a specific order but followed the flow of the conversation. Examples of main questions were, “In your experience, what role does ADHD play in sexual and reproductive health for young women?” and “Can you describe subjects concerning sexual and reproductive health that are raised by the women?” Probes and clarifying questions, such as, “Can you tell me more?” and, “Can you explain?” helped us to reach a deeper understanding. Both individual interviews and focus groups resulted in rich data that answered the study aim. Interviews were recorded digitally and lasted for 24–54 min (median 40 min). They were transcribed verbatim by KW and double-checked with data to ensure accuracy. After reflecting on the transcripts, the data collection was halted since no new information emerged, and the interviews yielded rich data. Recruitment and participation occurred between November 2019 and January 2022, with pauses from March to August 2020 and December to August 2021.

### Data analysis

**Fig. 1:**
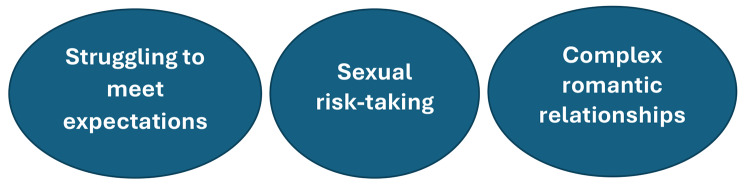
Final themes: HCPs’ perceptions and experiences of SRH in young women with ADHD

Data was analyzed using reflexive thematic analysis following the six phases described by Braun and Clarke [20, 21]. The method allowed the researchers to see patterns of shared meaning in the data and deepen understanding by identifying underlying ideas and assumptions. In the first phase, the researchers engaged with data transcripts by reading and re-reading, making notes of impressions and patterns. In the next two phases, all features answering the study aim were identified, and codes with similar meanings were organized into potential themes and subthemes. After this, in phase four, the themes were revised to make sure there were coherent patterns within them and clear distinctions between them, and that they reflected the whole data set. Within the revised themes, subthemes were found to cohere but to overlap, and the results were therefore only presented as themes. In the last two phases, informative names were constructed, and patterns and meanings of generated themes were defined and presented in a final thematic map (Fig. 1) and a written text.

To enhance research quality, the “15-point checklist for good thematic analysis” presented by Braun and Clarke guided the analysis [21]. For example, the researchers went back and forth between the six phases making sure that all the data was analyzed and interpreted, and that the themes fit the data. Reflexivity was integral to the process, prompting continuous questioning of code assumptions and interpretations and the researchers’ own influence on data analysis. Even though the researchers’ subjectivity was seen as a source for producing knowledge, they also acknowledged their preunderstandings in the research field to prevent overinterpretation [20]. The research team members further had knowledge and experience of the method used, which enhanced the credibility of the study [24]. To ensure that the experiences of the HCPs confirmed the study result, data analysis was carried out separately but was continuously discussed in the research team. Disagreements on themes were resolved by discussion in the group.

## Results

The data analysis resulted in three themes (Fig. 1). The results reflected the HCPs’ perceptions and experiences of SRH based on conversations with young women with ADHD in clinical meetings. Quotes are used to illustrate the results.

### Struggling to meet expectations

The HCPs described the women as like any young adults wishing to explore sexuality and romantic relationships. However, HCPs’ perception was that they did so in ways that were not always expected, breaking norms of sexual behaviors.

*“They have a behavior that we might not always expect, and the society and the structure around them might not be exactly modeled after these young women […] It’s just that they have a slightly different way of, like in a way, coping with themselves and the world.” Health care professional interview 1 (HCP 1)*.

Based on their perceptions and experiences, HCPs described the women to have feelings of not living up to expected behaviors, such as remembering to use contraceptives or choosing causal sexual partners more carefully, making some of them afraid of being judged for their actions and treated differently from others in health care settings. HCPs in youth clinics had experience of encountering women who did not want their ADHD diagnosis written in patient records or questioned why HCPs had to know about their diagnosis. HCPs interpreted that the women were afraid to be judged in advance.

*“She absolutely did not want me to write that she had a diagnosis […] She was afraid of being labeled in a certain way.” HCP 12*.

However, at youth clinics and women’s health clinics the women were perceived to as willing to discuss their sexuality openly. Most women were according to HCPs also eager to talk about their diagnosis, considering it an integral part of their identity that facilitated understanding during appointments. Conversely, in psychiatric clinics, where the diagnosis was already documented, HCPs’ perceptions and experiences indicated that some women avoided discussing sexuality unless specifically asked. The HCPs attributed this partly to women feeling ashamed of not meeting expected behavior, but also as a sensitive subject in general to raise for both the women and the HCPs. Nevertheless, when asked, they were often eager to discuss how symptoms of ADHD affected their sexual and romantic relationships.

The women were described by several HCPs as having positive sexual experiences because they dared to freely explore sexual relationships. However, even though some women were believed not to want to change their behavior, several others wished to fit in and conform to accepted norms. The HCPs’ perceptions and experiences indicated that most women were aware of sexual behavior expectations and knew which behaviors would benefit them, but struggled to live up to these expectations. The HCPs described encounters where women had blamed themselves for not being able to take care of themselves and their health as expected – forgetting contraceptives, missed health appointments, or rushing into sexual relationships they sometimes regretted.

*“The woman could say, ´I am that person who cannot have long relationships.’ Or ‘I am that person… who lets myself down again and again. Yes, I am hopeless. Now I forgot the condom again or I forgot my tablet. I am completely hopeless.’ Poor self-confidence results from this mindset.” HCP 13*.

As the women got older, expectations of being able to handle impulsive behavior, romantic relationships and structure in everyday life where also raised, which further reinforced self-criticism in the women, according to the HCPs. They had experience of women waiting to seek health care or finding it embarrassing to repeatedly come to test for STDs or pregnancy. Their interpretation was that some women felt ashamed or guilty because they had failed to live up to the believed expectations of sexual behavior. The HCPs’ perception was that accepting their view of themselves as persons who always failed could also make it easier for some women to seek health care without feeling ashamed.

*“But they can also end up with the fact that, ’I am this person. That’s just the way it is all the time.’ There will be an acceptance of being that person who always fails, always forgets the condom, and is always here testing herself.” HCP 14*.

### Sexual risk-taking

The HCPs’ perceptions and experiences indicated that curiosity, and fearlessness in engaging in new situations was considered resources for many of the women to learn more about their sexuality. However, HCPs perceived that the women’s difficulties in controlling impulses and understanding sexual boundaries or consequences of their actions could lead to sexual risk-taking. They described sexual risk-taking as sending explicit photos, having unprotected sex with multiple partners, and engaging in sexual encounters with unknown partners.

Individual differences in personality and functional abilities were highlighted by the HCPs to influence the degree of risk-taking. Having an ADHD diagnosis and medical treatment was perceived by the HCPs to help the women control impulses. Frequent visits to health care settings for STD and pregnancy testing or abortions were common among the women, according to the HCPs’ experiences.

*“It could be that you become curious and exploratory, daring to make contacts and experiment with desire. […] they often get a very positive response from others [….] but these young women with ADHD and with ADD, can become very impulsive in their relationship creation and, even then, sexually, have relationships in different ways, have sex, expose themselves to risks.” HCP 4*.

Acting in the first impulse to engage in sexual relationships could, according to the HCPs’ perceptions, carried a risk of ending up with partners they didn’t truly want to have sex with or engaging in sex in ways they weren’t comfortable with. HCPs described the women as often initiating sex or initially agreeing to it, making it challenging to change their minds during sexual activity. Regretting sex while engaged in it was perceived by HCPs as a risk of sexual assault because the women didn’t always feel comfortable saying no or felt they had the option to change their minds.

*“You throw yourself into something that you may not be completely comfortable with in retrospect. And that can lead to, I doesn’t need to be rape, but you can do things that you don’t really want, yet you don’t dare to say no […] I have met many women who describe having sex that felt right in the moment but uncomfortable afterwards.” HCP 10*.

Several HCPs described meeting women who had experienced sexual assault, including rape and sexual violence. They perceived the women to be at risk because they acted impulsively without taking time to reflect on the partner or being able to fully assess the partner’s intentions. Comorbidity with ASD further complicated partner assessment for the women, according to the HCPs, while emotional dysregulation with Borderline Personality Disorder (BPD) increased the risk of impulsive sexual behavior. The HCPs’ interpretation was that the women could become easy targets for sexual assault by offenders. A negative life spiral with poor mental health, missed school, and feelings of exclusion was also perceived by the HCPs as making the women more vulnerable to sexual offenders, as they sometimes sought attention and validation by unknown partners, engaged in sex to reduce stress or anxiety, or sold sex for drugs.

*“This woman also who had a very hard time understanding relationships and the intentions of others. She was also very lonely, with very few friends of her own and she felt grateful whenever someone gave attention to her. She absorbed the attention like a sponge.” HCP 16*.

### Complex romantic relationships

The HCPs described the women’s romantic relationships as complex. While some characteristics such as being social and unafraid to initiate contact, helped the women find partners, the HCPs’ perceptions and experiences indicated that they struggled to maintain and build healthy relationships. The HCPs perceived the women wishing for longer intimate relationships as they transcended from youth to young adulthood. However, unlike others, they didn’t always follow the typical pattern of settling down into long-term relationships and stable jobs. The women were described as searching for mutual love and intimacy, like other young adults, but according to HCPs, they often rushed into relationships, eager to fit in and find a sense of belonging.

*“Those I have met who desire a love relationship almost chase after it. They involve themselves, whether it’s good or not, because they seek some form of security. And then they are willing to do whatever it takes just to be in a relationship.” HCP 7*.

Some women were perceived by HCPs to at risk of exploitation or control by their partners, as they remained in relationships regardless of the cost. Fear of being rejected was interpreted by HCPs to be a result of low self-esteem, which further could reinforce the willingness to live up to the partners’ demands, such as agreeing to sex even when they didn’t want to. Even in relationships not considered destructive, according to HCPs, low self-esteem and uncertainty about managing relationships could hinder communication of wishes and desires due to fear of rejection and past experiences of not being listened to.

*“I think a lot of it was low self-esteem. She felt obligated to satisfy his needs, but she couldn’t ask him to satisfy her needs because then he would think that she was asking too much, that she was selfish, and he would want to leave her […]. Communicating her needs was very difficult for her.” HCP 11*.

Established romantic relationships, based on mutuality, were still perceived by the HCPs to be often complicated and characterized by conflict. Based on the HCPs’ experience, lack of focus could hinder the women from fully listening to a partner, leading to irritation and misunderstandings. Difficulties in regulating emotions sometimes resulted in hurtful comments, and a lack of impulse control could lead them to be unfaithful. According to HCPs’ experience, jealousy was common and could trigger impulsive reactions in the women, such as accusing their partners of being unfaithful, resulting in hurt feelings for both the women and the partners, and relationship break-ups. Some HCPs described the women as often being involved with partners who had similar diagnoses, which could make these relationships more prone to conflict.

*“Infidelity, I know, has come up, which feels involuntary really. That the woman is like, ‘I don’t really want to do this, but I’m with someone else sexually or sort of flirting with someone else. Then suddenly, I’m in different situations, and my partner reacts strongly to this, and I don’t really understand how this happened. It wasn’t on purpose to hurt my partner.’” HCP 4*.

The women’s life situation and management of ADHD symptoms were also described by HCPs as indirectly influencing the women’s relational conflicts and relational quality. General stress in the women’s lives was described as leading them to focus more on their own needs, spending less time with a partner and showing less interest in sex. Being aware of ADHD symptoms and how they may affect daily life for the women was further perceived by the HCPs to improve communication about needs and challenges in relationships. According to the HCPs, ADHD medication could also make it easier for the women to listen to a partner and manage daily chores, reducing conflict. Impulsive behavior and emotion regulation were also interpreted by the HCPs as being more easily controlled with medication.

‘When she takes medicine, she becomes calmer and feels that she has more energy to manage the relationship. […]’ Otherwise it takes so much energy for these girls when their thoughts go in and out and high and low all the time. But with medicine, they can feel calm and focus and have energy to have a dialog with their partner, because it is not spent on the ping pong balls in the head. HCP 10

## Discussion

The results of this study provide insights into HCPs’ perceptions and experiences of SRH in young women with ADHD. The women were perceived to struggle with managing expectations of sexual behaviors, possibly resulting in shame and fear of being judged by HCPs. HCPs acknowledged that sexual risk-taking behaviors were common among these women but varied based on functional ability, comorbidity, and life situation, sometimes leading to STDs and unplanned pregnancies. Challenges with impulse control, difficulties in assessing sexual partners and a wish to find validation from a partner were further interpreted as a possible risk for sexual victimization. The women were also perceived by HCPs to have a strong wish for longer relationships. While being social and unafraid were described to help the women meet partners, low self-esteem and the wish to fit in could lead to abusive relationships, and conflict could negatively influence relational quality. Emotion regulation and impulse control were perceived by HCPs to improve with ADHD medication, positively affecting relational quality for the women.

This study reveals that, based on HCPs’ perceptions and experiences, women with ADHD may feel ashamed of not meeting expectations related to sexual behavior. This shame was interpreted to be influenced by the women’s feelings of personal failure, self-labeling as irresponsible and an inability to take care of themselves. Our results show similarities with the study of Holthe and Langvik [25] who described how young women with ADHD attributed their perceived personal flaws to failure, leading to a subsequent risk of self-blame and a negative self-image. Our results may therefore indicate a risk for self-stigmatization, which according to Drapalski et al. [26] can result in reduced self-esteem and self-efficacy. According to Pender’s health behavior model [27] low self-esteem and self-efficacy could further be seen as a possible barrier for young women with ADHD to adopt healthy behaviors. Additionally, perceived shame may be exacerbated for the women in our study due to gender-related norms concerning sexual behavior [28]. For instance, Tholander and Tour [29] associated casual relationships with shame for young women in general, while Conley et al. [28] found that for young women compared to men, casual sexual relationships were less accepted by their peers. Holthe and Langvik [25] further emphasized the complexity of social norms in young women with ADHD by describing how symptoms of ADHD such as impulsiveness and energetic behaviors are less socially accepted among women. Hence, according to our results, HCPs in clinical settings need to be aware of the effect of stigma associated with both ADHD in young women and gendered social norms concerning sexuality.

An important finding in our study was that HCPs perceived that shame and fear of being judged of sexual behaviors could result in hesitation to seek health care or raise issues relating to SRH when meeting HCPs. This finding is strengthened by a study including young women, which demonstrated that anticipated judgment and shame could be a barrier to seek an appropriate level of health care after unprotected sex [30]. In youth clinics and gynecological clinics, HCPs described the women as openly discussing SRH but sometimes hesitating to disclose their diagnosis. This finding can be explained by previous research that revealed that adults with ADHD have experience of being stereotyped and labeled in health care settings, school and among peers [31]. In psychiatric clinics, both the women and HCPs hesitated to raise questions regarding SRH, possibly due to stigma related to SRH. However, it’s essential to recognize that our study explored HCPs perceptions and experiences. It is possible that questions about SRH were not raised by the women because there were no expectations to do so within the psychiatric context. Our study also described the women as speaking freely when asked about SRH in psychiatric clinics. This may indicate that the women are not sensitive about discussing this subject, rather, but it is the HCPs who feel uncomfortable bringing up the topic. In conclusion, HCPs need to have knowledge about ADHD and sexual behaviors regardless of their clinical specialization in order to create an environment where SRH can be openly discussed without judgment.

Sexual risk-taking behaviors presented in our study, such as having unprotected sex with several partners or with unknown partners, align with previous research on both young women and men with ADHD [10, 32, 33]. HCPs’ perceptions and experiences of the women’s sexual risk-taking behavior, as described in our study, may be partly explained by high sexual motivation and poor impulse control associated with normal development in young adults [7, 34]. However, it is likely to believe that impulse control in sexual situations is especially difficult for the women described by HCPs in our study. The high prevalence of executive dysfunction observed in individuals with ADHD [35] may challenge the women to control strong impulses to engage in sexual activities. Additionally, sexual risk-taking behaviors found in this study could be explained by research showing that individuals with ADHD are more prone to engage in activities resulting in immediate small rewards rather than delayed larger rewards [36]. The HCPs in our study also described that young women frequently visited clinics for STD and pregnancy testing or abortion counseling, consistent with studies showing a higher risk for STDs and unplanned pregnancies in women with ADHD compared to those without a diagnosis [10, 11]. Efforts to target contraceptive counseling toward young women with ADHD are therefore essential to prevent long-term medical and social consequences. However, our results also emphasized differences in personality and functional ability, suggesting that not all women exhibit risk-taking behavior. This accentuates the importance of considering each woman’s unique strengths and challenges during counseling. However, applying Pender’s health behavior model [27] engagement in healthy behaviors is not only influenced by personal characteristics. Counseling could therefore also be improved by exploring the women’s perceived benefits and barriers related to their sexual behaviors, allowing for a better understanding of their support needs.

Lack of impulse control or “doing what feels right in the moment”, according to HCPs in our study, resulted in positive sexual experiences for the women but with a risk of ending up in regretted or risky situations. Yet, our study revealed the risks faced by the women are multifaceted, not solely due to impulsive behavior. Comorbid diagnoses, life situation and difficulties to interpreting partner’s also play an important role. Acting on impulse, combined with not taking time to reflect on who the partner is or fully understand their intentions, nuances previous research, demonstrating a link between sexual risk-taking and sexual victimization in young women with ADHD [37]. Both challenges in detecting and interpreting social cues and lack of impulse control have be associated with ADHD [35, 38], but these challenges may according to our results be further amplified by ASD and BPD, suggesting that comorbidity may entail an additional risk for sexual regrets and sexual victimization. To build on suggestions made by Snyder [12], the women might, according to the HCPs, also be seen as easy targets by sexual offenders, because of their difficulty in assessing potentially risky situations. According to our results, a negative life spiral with poor mental health, missed school and feelings of exclusion could further enhance the women’s vulnerability to sexual offenders. Descriptions of seeking validation from unknown partners or engaging in sex to reduce stress or anxiety may reflect previous research revealing early peer rejection in girls with ADHD and an increased risk of self-harming behaviors in young adults with depression and anxiety [39].

This study reveals that HCPs’ perceptions and experiences indicated that young women with ADHD might engage and stay in romantic relationships even when these are not based on equal conditions. HCPs’ interpretation is that the women may stay in unhealthy relationships because they have a strong wish to have a relationship, or fear being rejected. These results may partly stem from societal pressure on emerging adults to conform to relationships expectations and low self-esteem resulting from earlier experiences of peer rejection [40]. Remaining in imbalanced relationships, where the partner holds decision-making power, risks relational dissatisfaction for young women with ADHD. Lower relational power is associated with less happiness, reduced trust in a partner, and decreased likelihood of having wishes fulfilled by a partner [41]. The lack of mutual trust and hesitation to communicate emotions and needs described in our study could further hinder intimacy in close relationships, leading to less satisfaction in sexual life.

According to the HCPs’ descriptions, stress and conflict could influence the relationship quality for these women. These findings are in accordance with Bruner et al. [14], who suggested that emotion regulation difficulties, perceived stress, and hostile relationship conflict may affect relationships in young women with ADHD. However, based on our results, the HCPs’ perception and experience indicated that ADHD medication and self-awareness might improve both conflict resolution and life management, strengthening previous studies suggesting benefits of early assessment and treatment of ADHD in young women [5]. Considering that lack of focus and emotional dysregulation pose communication challenges for the women described by HCPs in our study, involving partners in counseling may also enhance conflict resolution and their understanding of the women’s emotions and behaviors.

A strength of this study is that it is one of few focusing on HCPs’ perceptions and experiences regarding SRH in young women. One might argue that conclusions and interventions concerning SRH should be based mainly on the women’s own experiences. However, understanding HCPs’ views not only allows us to learn from their experiences but also provides us with a deeper understanding of one of two actors in health encounters. Expectations, perceptions and clinical context will most likely influence both women and HCPs in how they express themselves and thus also how support is provided. The diverse sample of HCPs in the study, including several different professionals from different types of clinics, further contributes to richer data, and a better understanding of SRH in young women with ADHD. The study further provides a clear description of the HCPs, the context in which the study took place and the research process in order for the reader to decide whether the result can be transferred to other contexts [22]. However, it is essential to recognize that this study reflects the HCPs’ perspectives. It is possible that the HCPs’ perceptions and experiences relate more to young women with severe functional disability, as they tend to seek more support from healthcare. Transferability can therefore be limited. However, the results offer deeper insights into challenges and risks, valuable for developing targeted counseling for women in greatest need of support. Future research could benefit from exploring the SRH support needs of young women with ADHD and develop interventions accordingly.

## Conclusions

According to HCPs’ perceptions and experiences, living with ADHD may lead to sexual risk-taking behaviors, resulting in regretted sex and sexual victimization, but it can also facilitate sexual exploration providing positive experiences and knowledge of sexuality for young women with ADHD. However, self-stigmatization and hesitation to discuss SRH issues with HCPs could be a risk for young women with ADHD, as they may not live up to perceived expectations of sexual behaviors. Additionally, symptoms of ADHD and secondary outcomes related to the diagnosis may challenge healthy qualitative relationships. The study emphasizes that HCPs need awareness of how comorbidity, life situation, stigma associated with ADHD and female sexuality, and individual variations in functional ability affect SRH. Regardless of their clinical expertise, HCPs should have knowledge about ADHD when counseling on SRH.

## Data Availability

The datasets generated and/or analyzed during the current study are not publicly available due to privacy concerns given the limited sample size but are available from the corresponding author on reasonable request and in accordance with the consent and restrictions of the ethical approval.

## Abbreviations

ADHD: Attention Deficit Hyperactivity Disorder
ASD: Autism Spectrum Disorder
BPD: Borderline Personality Disorder
SRH: Sexual and reproductive health
HCPs: Health care professionals
STD: Sexually transmitted disease
